# Plasticity in biomass allocation underlies tolerance to leaf damage in native and non-native populations of *Datura stramonium*

**DOI:** 10.1007/s00442-024-05585-0

**Published:** 2024-07-24

**Authors:** Franco Liñán-Vigo, Juan Núñez-Farfán

**Affiliations:** Laboratorio de Genética Ecológica y Evolución, Departamento de Ecología Evolutiva, Instituto de Ecología, Universidad Nacional Autónoma de México, Ciudad Universitaria, Circuito Exterior, 04510 Ciudad de Mexico, Mexico

**Keywords:** Defense, Enemy release, Fitness costs, Herbivory, Introduced populations

## Abstract

An introduction to a novel habitat represents a challenge to plants because they likely would face new interactions and possibly different physical context. When plant populations arrive to a new region free from herbivores, we can expect an evolutionary change in their defense level, although this may be contingent on the type of defense, resistance or tolerance, and cost of defense. Here, we addressed questions on the evolution of tolerance to damage in non-native Spanish populations of *Datura stramonium* by means of two comparative greenhouse experiments. We found differences in seed production, specific leaf area, and biomass allocation to stems and roots between ranges. Compared to the Mexican native populations of this species, non-native populations produced less seeds despite damage and allocate more biomass to roots and less to stems, and had higher specific leaf area values. Plasticity to leaf damage was similar between populations and no difference in tolerance to damage between native and non-native populations was detected. Costs for tolerance were detected in both regions. Two plasticity traits of leaves were associated with tolerance and were similar between regions. These results suggest that tolerance remains beneficial to plants in the non-native region despite it incurs in fitness costs and that damage by herbivores is low in the non-native region. The study of the underlying traits of tolerance can improve our understanding on the evolution of tolerance in novel environments, free from plants’ specialist herbivores.

**Supplementary Information:**

The online version contains supplementary material available at 10.1007/s00442-024-05585-0.

## Introduction

When plants are introduced into a new range, where biotic and abiotic conditions change, the beneficial role of plant defenses might change. It has been proposed that changes in plants’ phenotypic plasticity, competitive ability, and defense can play a role in the successful establishment of colonizing populations (Yeh and Price 2004; Drenovsky et al. 2012a; Erfmeier 2013). A first benefit envisaged for plants from an introduction to a new habitat is their release from defense-adapted enemies, increasing its survival rates and promoting positive population growth rates, enabling their establishment, and even outperforming native plants (Keane and Crawley 2002). This scenario corresponds to the enemy release hypothesis (ERH). According to this scenario, it would be expected an evolutionary change in plant defense, since it uses resources from the plant that may be channeled to other functions (Herms and Mattson 1992). The evolution of increased competitive ability (EICA) hypothesis suggests that non-native, introduced plants, would make a shift in plant defense investment, toward investments of resources to enhance growth, reproduction, and competitive ability (Blossey and Nötzold 1995). There is empirical evidence for reduced levels of defense in introduced populations (Lin et al. 2015), supporting the expectation of the EICA hypothesis. However, empirical evidence likewise documents cases of no change in this ability (Van Kleunen and Schmid 2003; Bossdorf et al. 2004) or even increased defense levels in introduced populations (Stastny et al. 2005; Ashton and Lerdau 2008). These mixed results suggest complex scenarios for the evolution of defense after introduction to a new range.

Defenses are conformed by a suite of characters that prevent damage from herbivores (resistance) or buffer the negative effects of it on fitness (tolerance) (Stowe et al. 2000; Núñez-Farfán et al. 2007). Traits commonly associated with resistance are chemical compounds and physical barriers like trichomes (Shonle and Bergelson 2000; Valverde et al. 2001), whereas traits associated with tolerance are traits related to growth, biomass allocation, photosynthetic activity, and plastic responses after damage (Strauss and Agrawal 1999, Stowe et al. 2000; Tiffin 2000). These traits can involve costs for plants, in the form of allocational costs (fitness cost in the absence of herbivores) (Simms and Triplett 1994; Mauricio et al. 1997), ecological costs (when defense to one herbivore affects defense to another herbivore or affects a positive interactions like pollination) (Pilson 1996; Strauss et al. 2002), or a trade-off between resistance and tolerance (a genetically negative correlation between resistance and tolerance) (van der Meijden et al. 1988; Fineblum and Rausher 1995; Leimu and Koricheva 2006; Oduor et al. 2011). Since defenses incur in cost, a reduction in levels of resistance or tolerance is expected when herbivores are absent, nonetheless, the maintenance or increase in a defense can be possible if traits that confer defense play roles other than defense, mitigating costs (Herms and Mattson 1992; Fornoni 2011). For example, tolerance traits can increase competitive ability (Zou et al. 2008) or be genetically correlated with growth (Weis et al. 2000; Camargo et al. 2015; Avila-Sakar 2020); if so, selection favoring competitive ability or growth in the new range, may favor tolerance too (Uriarte et al. 2002; Ashton and Lerdau 2008; Fornoni 2011). On the other hand, if resistance traits, like chemical compounds, are reduced in the new range, and there is a trade-off between resistance and tolerance, we can expect a shift in the type of defense, favoring tolerance as predicted by ecological theory (Rosenthal and Kotanen 1994; Fineblum and Rausher 1995; Stastny et al. 2005).

Since trade-offs between resistance and tolerance are expected in plants (Leimu and Koricheva 2006), a reduction in the level of resistance would suggest an increase in tolerance in an environment free from specialist herbivores, product of a reallocation of resources spent to herbivore resistance toward growth and reproduction that can contribute to increase tolerance (Jones et al. 2006; Huang et al. 2010). In the self-compatible annual *Datura stramonium*, it has been found reduced levels of alkaloids (resistance traits) in introduced populations (Castillo et al. 2019) and these populations experience less damage and are larger than native populations (Valverde et al. 2015) suggesting potential changes in the levels of defense, enhanced growth and/or competitive ability. Under this scenario, it is likely that tolerance had increased in introduced populations relative to native populations of *D. stramonium*.

Here, we examine the phenotypic differences in vegetative traits between native and introduced populations of *Datura stramonium* as well as the differences in phenotypic response to leaf damage, specifically, fitness tolerance to leaf damage. Also, we investigated the association between vegetative traits and the capacity to tolerate leaf damage in these populations, to answer the following questions: Do native and non-native populations of *D. stramonium* differ in vegetative traits and seed production? Do native and non-native populations respond different to leaf damage and, if so, do this differential response to leaf damage affects tolerance?

## Materials and methods

### Study system

*Datura stramonium* L. (Solanaceae) is a cosmopolitan annual self-compatible weed, native to North America, Mexico and south United States, where it can be found in a wide array of plant communities (Weaver and Warwick 1984; Núñez-Farfán and Dirzo 1994). In Spain, *D. stramonium* was likely introduced after the conquest of Mexico, between 1540 and 1577. This plant occurs in agricultural fields, riverbanks, roadsides, and other ruderal habitats (Sanz-Elorza et al. 2005). The level of leaf damage produced by arthropod herbivores is different between regions (native/non-native); the Spanish populations receive a lower mean proportion of leaf damage than Mexican populations (2.5% and 50.3%, respectively; Valverde et al. 2015). In Mexico, the beetles *Lema daturaphila* and *Epitrix parvula* (Coleoptera: Chrysomelidae) are specialist consumers of the leaves of *D. stramonium* (Núñez-Farfán and Dirzo 1994). In Spain, these beetles are absent and the generalist herbivore, *Helicoverpa armigera* (Noctuidae: Lepidoptera), consumes the leaves of* D. stramonium* (Valverde et al. 2015).

### Greenhouse experiment

We performed two greenhouse experiments. The first experiment included two populations from each origin (Spain and Mexico), each population with 15–20 families (full siblings). From the native region of *D. stramonium* in Mexico, we selected the localities of Teotihuacán and Ticumán, whereas in the non-native range in Spain, we assessed plants from the localities of Valdeflores and La Zubia (Zubia, hereafter), both in Andalusia, Spain. Plants were germinated and transferred into individual pots filled with sandy field soil and distributed at random to benches in the greenhouse. We assigned two to four plants to each damage treatment for a total of 483 plants. Two levels of damage were used, 0% and 50% of leaf area removed. This experiment was performed in a greenhouse of Universidad Autónoma de Chapingo, Texcoco, México.

In the second experiment, we used 10 families (natural progenies), randomly selected from each population (same populations from the first experiment), and three levels of simulated leaf damage. Seeds were germinated into Petri dishes in environmental chambers at 28/23 °C with a 12:12 light/darkness photoperiod. The seed coat was removed to enhance germination. When the cotyledons were fully expanded (between the seven and ten day), plants were transplanted to individual pots with a mix of 3:2 sand and perlite and placed at the greenhouse and distributed in a random fashion to avoid environmental heterogeneity inside the greenhouse. Nutrients were added as a 100 mL solution of Peter’s® 20-20-20 NPK at a concentration of 2 g/L to all pots weekly for five weeks. There were ca. 3–4 plants per family randomly assigned to each of the three levels of damage (0%, 30%, 60% of leaf area removed) making a total of 444 plants. This experiment was performed in the greenhouse of Instituto de Ecología, UNAM, CDMX, México.

The substrate used in the first experiment, field sandy soil, had a natural and uncontrolled source of nutrients which in turn can mask differences in biomass allocation between plants of different regions. Therefore, for a better assessment of biomass allocation, the concentration of nutrients in the second experiment was controlled using a combination of sand and perlite where plants do not grow unless nutrients are added. Conducting greenhouse experiments was preferred over natural conditions to achieve better control in the amount of damage that plants receive since amount of damage received by the plants can be a random variable but also can be the result of plant resistance (constitutive or/and induced). A previous study (Núñez-Farfán et al. 2024) found that the populations used in this study differed in the level of infestation and damage in field which can bias the tolerance estimation. We can avoid these confounding variables by applying fixed levels of damage in a greenhouse.

We applied damage at the same phenostage for all plants in both experiments, (i.e., the reproductive stage of these plants) at which the herbivore consumption is higher (Núñez-Farfán and Dirzo 1994; Fornoni and Núñez-Farfán 2000), simulating the damage made by the leaf beetles (i.e., holes on the leaf blade without damaging the principal veins) using a cork-borer. Before applying damage, we measured the leaf area of each plant using the next equation: leaf area = 2.47*leaf length + 0.34*leaf length^2^ − 12.37 (*R*^2^ = 0.97, *n* = 30). At the end of the experiments (thirty and sixty days after application of damage for the first and second experiments, respectively), we measured, for each plant, the total leaf area, seed number, seed mass and seed size. The plants were separated into leaves, stems, and roots to obtain their dry mass and were divided by total dry mass (total biomass) in the calculation of leaf mass fraction (LMF), stem mass fraction (SMF) and root mass fraction (RMF). In the first experiment, reproductive effort was measure as the dry weight of reproductive structures divided by total dry mass. The specific leaf area (SLA) was measured as the area of a fully expanded leaf and divided by its dry weight in the second experiment.

### Data analysis

All data were analyzed using R version 4.1.3 (R Core Team 2022).

### Phenotypic differences

To test for differences between regions and damage levels, we fitted hierarchical mixed-effects linear models for each trait with the *nlme* package (Pinheiro and Bates 2022). Region (Mexico vs. Spain), damage, and the interaction between region and damage, were considered as fixed effects and populations nested within region and families nested within population as random effects. The plant´s leaf area before the application of damage was used as a covariate to adjust for size of the plant. The significance of fixed effects was calculated with a Wald’s chi-square test and the significance of random terms was calculated by comparing models with and without the term, using likelihood-ratio tests (Zuur et al. 2009; Fox and Weisberg 2019). Before analyzing, vegetative allocation traits from the first experiment were arcsine transformed while total biomass of the second experiment log transformed to ensure assumptions of normality and homoscedasticity. Comparisons between levels of damage in the second experiment were made with Tukey’s post hoc test for least square means.

### Plasticity and tolerance estimation

We calculated plasticity for all traits as the slope of the family mean trait value as a function of damage. The tolerance estimation for each family corresponds to the slope of the family mean seed number on damage. This approach treats tolerance as the fitness response (or plasticity) to an environmental factor, in this case, leaf damage (Simms 2000). With these measurements we tested for differences in plasticity between regions using a nested ANOVA, with populations nested in regions.

### Cost of tolerance

The fitness cost of tolerance was estimated as the family mean correlation between the tolerance measure and fitness (seed number) in absence of damage (Simms and Triplett 1994; Mauricio et al. 1997). A significant negative correlation coefficient can be considered as evidence for fitness cost of tolerance. We calculated the cost of tolerance for each region and coefficients were compared with a Fisher’s *z* transformation to test for differences in cost between regions using the *cocor* package (Diedenhofen and Musch 2015).

### Traits related to tolerance

To assess relationships between tolerance and traits measured in both experiments, we used a stepwise multiple regression approach (Strauss et al. 2003; Wise et al. 2008). The response variable was tolerance, and the predictors variables were plasticity values of vegetative traits and average value of vegetative traits (family means) measured in undamaged plants. Vegetative traits included in the model were the traits that resulted related to fitness in the absence of damage after a previous stepwise regression. In the previous stepwise procedure seed number in the absence of damage was the response variable and the mean values of vegetative traits in the absence of damage were the predictors (Table S3). The variables were added at an entrance level of *P* = 0.15 and kept in the model at the “stay” level of *P* = 0.10. The analysis was run for each region and combining them.

## Results

### Region and damage effects

**Fig. 1:**
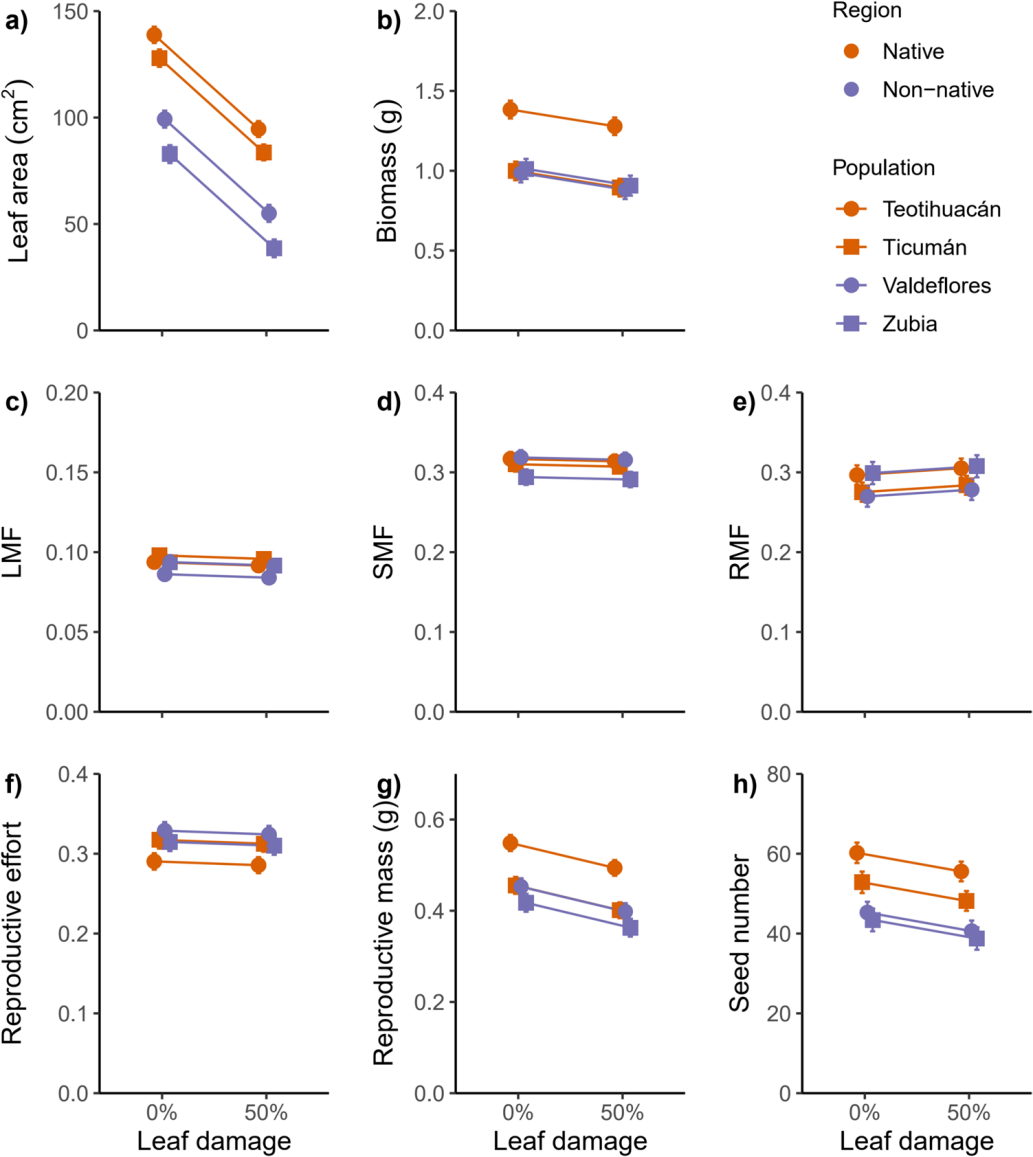
Phenotypic responses of native and non-native populations of *Datura stramonium* to leaf damage in the first experiment. Native Mexican populations: Teotihuacán and Ticumán, non-native Spanish populations: Valdeflores and Zubia. Least squares means and one standard error are represented. *LMF* leaf mass fraction, *SMF* stem mass fraction and *RMF* root mass fraction

In the first experiment, non-native populations had 38.8% less leaf area than native populations (*χ*^2^ = 16.81, *P* < 0.0001) across damage levels (Fig. 1A). Total biomass was not different between regions (*χ*^2^ = 1.75, *P* = 0.19), although there were differences between populations within regions (*χ*^2^ = 52.96, *P* < 0.001), and significantly decreased with damage in all populations (*χ*^2^ = 14.776, *P* = 0.0001) (Fig. 1B). Region had a non-significant effect on biomass allocation to leaves, stems and roots: LMF (*χ*^2^ = 2.11, *P* = 0.15), SMF (*χ*^2^ = 0.22, *P* = 0.64) and RMF (*χ*^2^ = 0.061, *P* = 0.80, respectively). Damage also had a non-significant effect on these traits: LMF (*χ*^2^ = 0.35, *P* = 0.56), SMF (*χ*^2^ = 0.25, *P* = 0.62), and RMF (*χ*^2^ = 1.27, *P* = 0.26). The reproductive effort was approximately 5% higher in non-native populations in the damage treatment but this difference was not significant (*χ*^2^ = 1.22, *P* = 0.27), although populations within region had a significant effect (*χ*^2^ = 6.52, *P* = 0.0053) (Fig. 1F). As with the allocation to vegetative parts, damage had a non-significant effect on reproductive effort (*χ*^2^ = 0.25, *P* = 0.62). The seed number significantly differed between non-native and native populations (*χ*^2^ = 10.29, *P* = 0.0013), where non-native populations produced 22.8% less seeds than native populations. Damage decreased the production of seeds (*χ*^2^ = 6.75 *P* = 0.0094) and the interaction region × damage was non-significant (*χ*^2^ = 0.19, *P* = 0.66) (Fig. 1H), suggesting no difference in tolerance to leaf damage of non-native and native populations.

**Fig. 2:**
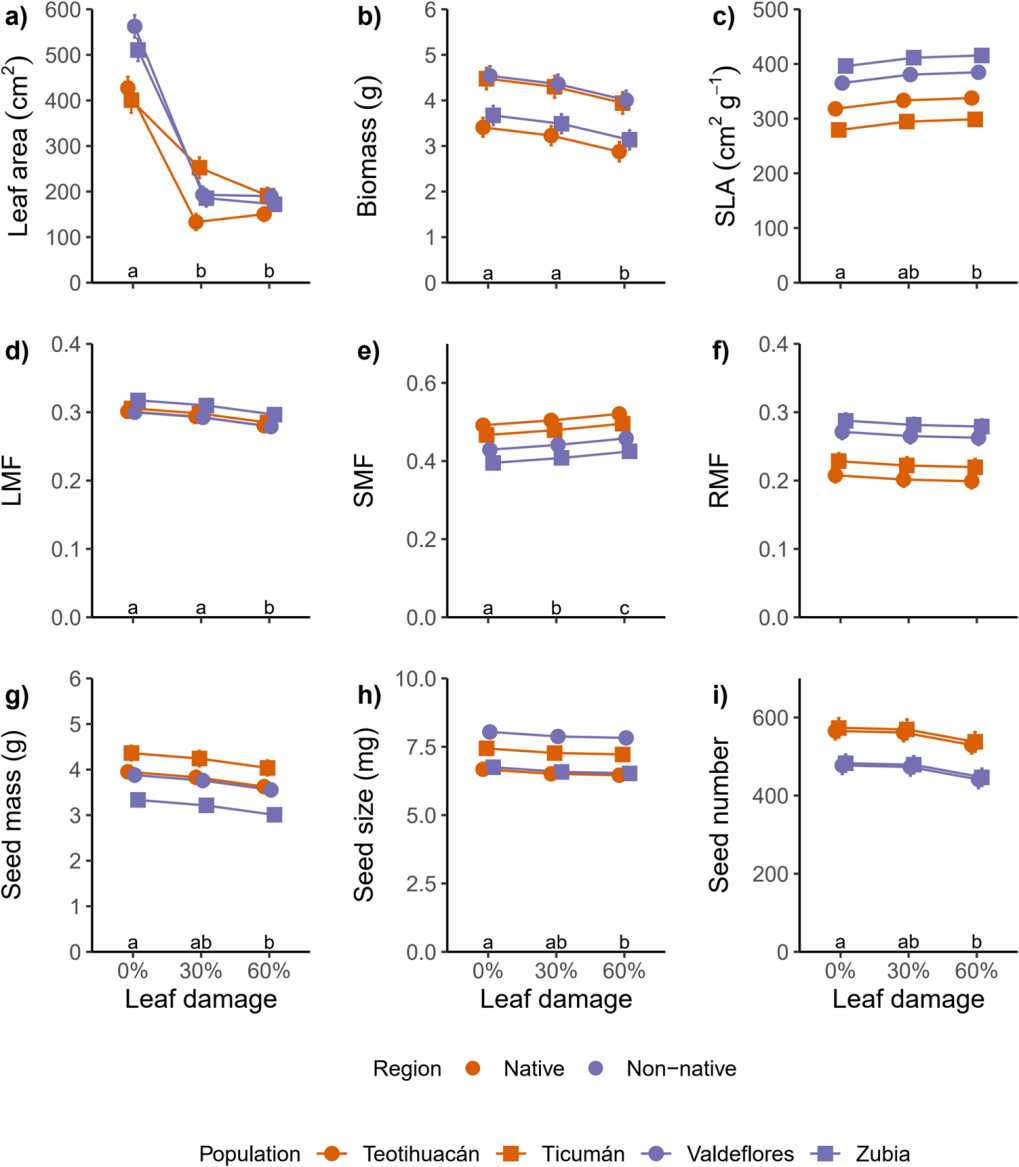
Phenotypic responses of native and non-native populations of *D. stramonium* to leaf damage for the second experiment. Native Mexican populations: Teotihuacán and Ticumán, non-native Spanish populations: Valdeflores and Zubia. Least square means and one standard error are represented. Letters indicate significant differences between levels of damage. *LMF* leaf mass fraction, *SMF* stem mass fraction and *RMF* root mass fraction

In the second experiment, non-native populations had 14% more leaf area than native populations across damage levels (Fig. 2A). Damage significantly reduced leaf area (*χ*^2^ = 279.51, *P* < 0.001) and the interaction region × damage was significant (*χ*^2^ = 8.048, *P* < 0.018). There was no difference in total biomass between native and non-native populations (*χ*^2^ = 0.083, *P* = 0.77), although there was a significant effect of population within region (*χ*^2^ = 83.88, *P* < 0.0001) (Fig. 2B) and damage significantly reduced the biomass (*χ*^2^ = 36.085, *P* < 0.0001) of both native and non-native populations. The specific leaf area (SLA) of non-native populations was 25% higher than native populations (*χ*^2^ = 10.52, *P* = 0.0012), and damage marginally increased the SLA of all populations (*χ*^2^ = 5.64, *P* = 0.059 and *χ*^2^ = 3.233, *P* = 0.198 for the interaction region × damage) (Fig. 2C). The leaf mass fraction was not significantly different between native and non-native populations (*χ*^2^ = 0.33, *P* = 0.57) and damage reduced the LMF (*χ*^2^ = 22.23, *P* < 0001) of all populations. The SMF was 13.7% lower in non-native populations (Fig. 2E), while the RMF was 29.2% higher in non-native populations than native plants (Fig. 2F), and there was a significant effect of damage only on the SMF (*χ*^2^ = 35.58, *P* < 0001) for both regions. The seed mass and seed size decreased with damage (*χ*^2^ = 11.48, *P* = 0.0032 and *χ*^2^ = 6.72, *P* = 0.034, respectively), there was no significant difference between regions for these traits (*χ*^2^ = 2.44, *P* = 0.12 and *χ*^2^ = 0.17, *P* = 0.68, respectively) although there was a significant population effect for both traits (*χ*^2^ = 23.32, *P* < 0.0001 and *χ*^2^ = 113.08, *P* < 0.0001, respectively) (Fig. 2G and 2H). Seed number was 15.7% lower in non-native populations (*χ*^2^ = 16.09, *P* < 0001) and decreased with damage (*χ*^2^ = 8.36, *P* = 0.015) (Fig. 2I). We did not detect a significant interaction region × damage (*χ*^2^ = 0.503, *P* = 0.78) suggesting similar levels of tolerance of native and non-native populations as in the first experiment.

### Plasticity in native and non-native populations

**Fig. 3:**
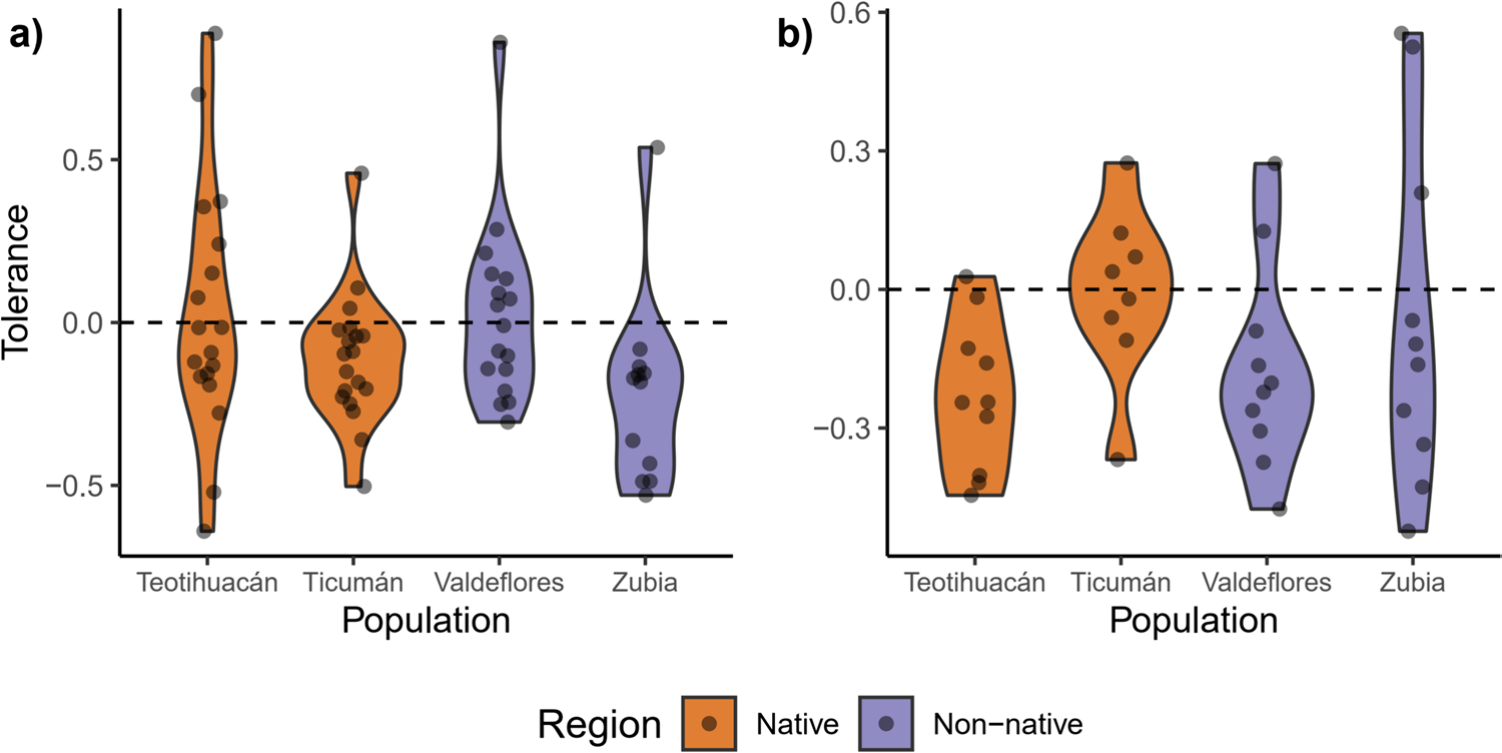
Tolerance values (as a measurement of plasticity in seed number) for native Mexican populations and non-native Spanish populations of *D. stramonium*. **A** values for the first experiment. **B** values for the second experiment

In both experiments, native and non-native populations had a similar response to damage since there were no differences in plasticity for most traits (Table S1 and Table S2). Plasticity in leaf area was significantly different between regions, although only marginally in the second experiment. Native populations had slopes less negative than non-native populations, suggesting less pronounced reductions in leaf area at the end of the experiment. Tolerance measured as the plasticity of the seed number was not significantly different between regions in both experiments (Fig. 3).

### Fitness cost of tolerance

**Fig. 4:**
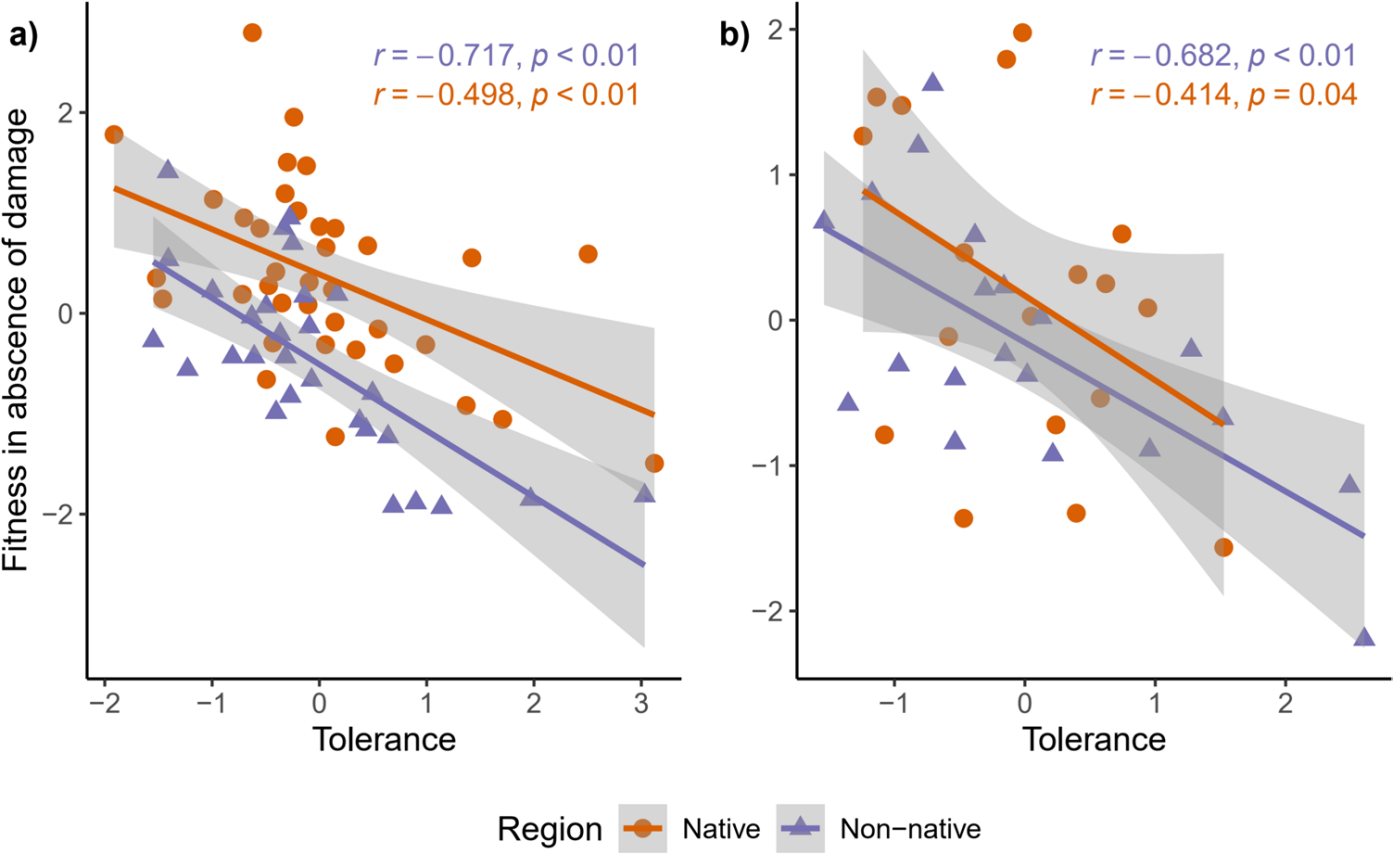
Fitness cost of tolerance. **A** Experiment 1. **B** Experiment 2. Fitness costs were calculated for each region. Points represent family means values

In both experiments, we detected a significant negative correlation between tolerance and fitness (seed number) in absence of damage for both regions, suggesting the existence of fitness cost of tolerance in both regions (Fig. 4). Although the negative correlation in non-native populations is higher than native population, there was no significant difference between coefficients (Table S4). It must be noted that native populations had a larger intercept value than non-native populations, indicating a higher seed number in native populations and this result match with previous analysis (Figs. 1H and 2I).

### Traits associated with tolerance

We found two plasticity traits associated with tolerance. In the first experiment, these were plasticity in biomass and plasticity in leaf mass fraction (LMF) (negatively); while in the second experiment, plasticity in leaf area and plasticity in LMF (negatively) were associated with tolerance (Table 1). The same analysis, within each region, found different traits associated with tolerance. For the first experiment, plasticity in LMF (biomass allocation to leaves) was negatively associated with tolerance in native populations (12% of variation explained), whereas plasticity in biomass and plasticity in SMF were associated positively with tolerance in non-native populations (51.7% of variation explained). In the second experiment, two traits were related to tolerance in native populations, plasticity in biomass and biomass allocation to stems (SMF) (negatively associated) (45.3% of variation explained). Traits related to tolerance in non-native populations were leaf area and plasticity in LMF (negatively associated) (38.5% of variation explained).

**Table 1:** Traits related to tolerance to leaf damage in *D. stramonium*

	Region	Trait	Estimate	Cum. *R*^2^	*P*-value
Experiment 1		Plasticity in biomass	0.326	0.182	0.0104
		Plasticity in LMF	− 0.249	0.219	0.0484
Experiment 2		Plasticity in leaf area	0.527	0.069	0.0017
		Plasticity in LMF	− 0.514	0.271	0.0022
Experiment 1	Native	Plasticity in LMF	− 0.386	0.119	0.0205
	Non-native	Plasticity in biomass	0.635	0.416	0.0036
		Plasticity in SMF	0.421	0.517	< 0.0001
Experiment 2	Native	SMF	− 0.425	0.283	0.0078
		Plasticity in biomass	0.345	0.453	0.0272
	Non-native	Leaf area	− 0.909	0.149	0.0067
		Plasticity in LMF	− 0.745	0.385	0.0119

The relationships were estimated combining regions and for each region after a stepwise procedure

## Discussion

Plants introduced into a new range and free from their natural (herbivores) enemies are expected to divert resources saved from a costly antiherbivore defense (as resistance) toward an increase in competitive ability, growth, and reproduction which in turn can contribute to tolerance to herbivores. Here, we demonstrated experimentally that tolerance to leaf damage in *D. stramonium* remains equivalent between populations of *D. stramonium* from Mexico (native) and Spain (non-native), but they do differ in allocation patterns (underlying traits of tolerance), and allocation to fitness-related traits.

### Phenotypic differences between regions

We detected differences in phenotypic traits in non-native and native populations of *Datura stramonium,* in both experiments, that likely result from genetic differentiation between these populations. Native populations produced more seeds than non-native population, despite the level of leaf damage, in both experiments (Figs. 1H and 2I) and seed mass and size were not different between regions in the second experiment (Fig. 2G and H). A higher production of seeds can be attributed to the amount of leaf area available to produce photosynthates. In the first experiment, native populations had more leaf area than non-native populations (Fig. 1A), and this difference in leaf area can account for the higher seed production in native populations in comparison to non-native populations of *D. stramonium*, since there is a positive relationship between seed number and leaf area (Table S3)*.* Nonetheless, in the second experiment, non-native populations were the ones that have more leaf area than the native populations, although only in the absence of damage (Fig. 2A), and the native populations again produced more seeds than non-native populations, suggesting that the production of seeds in *D. stramonium* is not only determined by the amount of leaf area available. The difference in final leaf area can also arise from the fact that the amount of time spanned after the application of damage was twofold larger in the second experiment than in the first experiment (60 days and 30 days, respectively), and non-native populations had more time to produce new foliar tissue after the application of damage until the end of experiment. Also, in the second experiment, plants had a longer period to recover for fruit and seed development since the application of damage. This result enables us to determine that the differences in seed production between plants of the two ranges holds despite the difference in time available to produce seeds between experiments (Figs. 1H and 2I).

The biomass allocation to roots and stems were significantly different between populations although only in the second experiment. Since in the second experiment the level of nutrients in the substrate was controlled, true differences in biomass allocation between native and non-native populations were detected. Native populations allocated more biomass to stems and less to roots (higher SMF and lower RMF), but the inverse pattern was found in non-native populations (Fig. 2E and F) and allocation to leaves was not different between regions (Fig. 2D). This result suggests a shift in allocation priorities in the non-native populations. A similar result where the non-native populations allocate more biomass to roots was found for *Climedia hirta* (DeWalt et al. 2004), *Senecio inaequidens* (Bossdorf et al. 2008) and when comparing introduced to native species of *Solidago* (Meyer and Hull-Sanders 2008; Szymura and Szymura 2015) and *Acer* (Morrison and Mauck 2007). Shifts in biomass allocation are expected when plants are moved to a new habitat since can enhance competitive ability, favoring their establishment (Egli and Schmid 2000). Higher allocation to roots can contribute to the storage of resources enhancing reproduction in a new habitat. However, non-native populations produced less seeds than native populations of *D. stramonium*. More biomass allocated to roots can enhance the capture of limiting resources in soil like nutrients or water and this can result in an enhanced competitive ability (Aerts et al. 1991; Chapin et al. 1993; Grotkopp and Rejmanek 2007; Poorter et al. 2012; Shang et al. 2015). This can enable *D. stramonium* to compete for such resources in water or nutrient-limited environment in non-native region (DeWalt et al. 2004; Grotkopp and Rejmanek 2007), but reducing the production of seeds in comparison to native counterparts since it is possible that an enhanced competitive ability in vegetative stage comes with a fitness cost. In Mexico, *D. stramonium* germinates in the warm rainy season; while in Spain (the non-native habitat), it germinates in the hottest and drier season where resources like water can be limited. Under these conditions, an increased allocation to roots can be beneficial enabling plants a maximize resource capture (Jiménez-Lobato et al. 2018). Less biomass allocation to roots has been found for other species in a non-native habitat, like *Jacobea vulgaris* (Lin et al. 2015). The native genotypes of *J. vulgaris* have shown increased allocation to roots, which is related to their ability to regrowth, a component of tolerance in this species, suggesting a shift in resource allocation of non-native genotypes. It seems that prioritizing shoot allocation increases potential growth at expenses of tolerance. Here, we found no differences between native and non-native plants of *D. stramonium* in tolerance, so it is likely that differences in root biomass allocation between native and non-native plants do not necessarily reflects differences in tolerance in *D. stramonium*. This highlights that tolerance can rely on different traits for different species and that the allocation of biomass to roots can be influenced by other factors in a non-native region such as soil condition or local competition (Tiffin 2000; Poorter et al. 2012).

Furthermore, in addition to increased allocation to roots, non-native populations of *D. stramonium* displayed higher values of SLA. Several studies have found higher values of SLA for non-native populations (Meyer and Hull-Sanders 2008; Feng et al. 2008; Drenovsky et al. 2012b; Gard et al. 2013) and this can contribute to the success in the establishment of non-native populations in novel habitats since this trait is positively related with relative growth rate (RGR) (Poorter and Remkes 1990; Poorter et al. 2009). Higher value of SLA can be translated in higher values of RGR, nonetheless, SLA it is not the only component of relative growth rate. Another component, the net assimilation rate (NAR), can influence the variation in RGR. There is a negative relationship between NAR and SLA (Poorter and van der Werf 1998), thus increases in one of these components does not necessarily translate in an increase in RGR. In our system, the difference in SLA may explain, partially, the fact that non-native populations had more final leaf area as well as leaf size (Figure S2), than native populations in the second experiment. In non-native populations, high SLA values imply more leaf area for unit of leaf mass, suggesting low constructional costs of leaves at expenses of photosynthetic capacity (Poorter 2002; Feng et al. 2008). This in turn reduces plant resources available to produce seeds.

### Phenotypic plasticity

Phenotypic plasticity of vegetative traits in response to leaf damage is an important component of tolerance to this stress (Wise and Mudrak 2023) and it has been considered as a crucial factor facilitating the colonization of novel habitats by introduced populations and species. For this reason, it is expected that introduced populations would express higher levels of plasticity in comparison to native populations (Yeh and Price 2004; Richards et al. 2006; Drenovsky et al. 2012a). However, this was not the case for non-native populations of *D. stramonium* (Fig. 3) since there were no significant differences in plasticity between native and non-native populations for most traits. There was a significant difference of plasticity for leaf area in the first experiment and marginally significant in the second experiment (Table S3). The difference in plasticity for leaf area was in favor of native populations (Figure S1). This result indicates that the ability to compensate foliar tissue in response to damage is lower in non-native populations (Fig. 2A). The absence of difference for plasticity in traits related to biomass allocation suggest that biomass allocation patterns between native and non-native populations are affected in a similar fashion by foliar damage, and that damage produced slight changes in allocation in both experiments (Figs. 1 and 2). There is evidence that trait plasticity may not be the main difference between native and introduced populations (Godoy et al. 2012; Matzek 2012; Marchini et al. 2019), since some studies found similar levels or even reduced levels of phenotypic plasticity for introduced populations (Drenovsky et al. 2012b; Hirsch et al. 2016; Kleine et al. 2017; Marchini et al. 2019). The principal difference between native and non-native populations may be the size of trait values instead of the magnitude of its plasticity (Godoy et al. 2012; Matzek 2012) and this difference in trait values is what enables introduced plants to colonize novel habitats. This may be true for non-native populations of *D. stramonium* since they differ in trait values for the SLA, and allocation to stems and roots in comparison to native populations. This combination of traits may enable survival and resource capture in habitats with a Mediterranean climate and associated with the enhancement of competitive ability in such habitats (Grotkopp and Rejmanek 2007). More efficient resource capture can be very important for non-native populations of *D. stramonium* since its reproductive phase occurs in the hottest and driest period in Spain (Jiménez-Lobato et al. 2018). Plasticity can have a role in early phases of colonization of novel habitats and after, establishment of the populations, the high levels of phenotypic plasticity can be reduced due to the potential maintenance costs of plasticity (Palacio-López and Gianoli 2011; Lande 2015). This scenario can contribute to explain why some studies find higher levels of plasticity in introduced populations, and that the time since their introduction is a factor to consider when comparing native and non-native populations.

Another aspect of plasticity that is noteworthy to consider is that it is both trait specific and environmental factor specific. Therefore, the capacity of expressing a plastic response is contingent with the focal trait and the environmental factor of interest (Bradshaw 1965; Wise and Mudrak 2023). Studies that report differences in plasticity for non-native and native populations had evaluated the plasticity in response to abiotic factors like nutrients, water, or pH (Kaufman and Smouse 2001; Bossdorf et al. 2008; Caño et al. 2008; Funk 2008; Shang et al. 2015). In contrast, we failed to detect differences in the phenotypic plasticity for a varying biotic environmental factor, leaf damage, between native and non-native populations. Although these results do not rule out higher phenotypic plasticity in non-native populations in relation to native ones, it might be in response to other environmental factors either abiotic or abiotic or to their interaction. Nonetheless, further experiments are badly needed to determine whether non-native populations have higher levels of phenotypic plasticity on defense-related traits, and when abiotic factor varies simultaneously (e.g., soil nutrients).

### Tolerance to leaf damage in non-native populations

In a novel habitat free from specialist herbivores, it is expected that plants would reduce its defense levels in favor of growth and competitive ability, as suggested by the EICA hypothesis. Our results do not support the EICA hypothesis with respect to defense levels. Non-native populations of *D. stramonium* displayed similar levels of tolerance to leaf damage; seed production was reduced in similar fashion in native populations and non-native populations (Figs. 1H, 2 and 3). When considering other traits than seed production as fitness measure, like biomass, seed mass, and seed size, the results are similar (Fig. 2B, G and H); plants for both regions get reduced their seed production in number and mass under leaf damage. A limitation of this study was that we attempted to mimic natural herbivory by applying mechanical damage, and it is unknown whether plant response, in the measured traits, would differ between types of damage. Nonetheless, the application of simulated herbivory was preferred to ensure that all plants receive the same amount of damage despite their level of resistance, since there is evidence that these plants differ in resistance levels (Valverde et al. 2015; Castillo et al. 2019), which can result in different herbivore consumption and masking differences in tolerance (Tiffin and Inouye 2000; Waterman et al. 2019). Additionally, when studying tolerance and growth-related traits, simulated and natural damage often shows similar effects, and this is better achieved when the spatial pattern and timing of application are imitated (Lehtilä and Boalt 2008; Waterman et al. 2019). We followed these rules to apply leaf damage in these experiments (see Greenhouse experiment section).

Similar tolerance levels between native and non-native populations have been found for *Alliaria petioliata*, *Solidago canadensis,* and *Ambrosia artemissifolia* (Van Kleunen and Schmid 2003; Bossdorf et al. 2004; Gard et al. 2013). One possible argument is that of “not enough time” has passed since introduction as to promote further evolution of different levels of tolerance. However, considering that *D. stramonium* was introduced into Spain in the XVI^.^ century from Mexico, it seems that the not enough time argument would not apply for non-native *D. stramonium* populations in Spain (Prentis et al. 2008). However, *D. stramonium* could have been introduced repeatedly into Europe and this continuous “gene flow” would prevent differentiation in relation to native populations (Müller-Schärer and Steinger 2004). A preliminary genetic analysis of the non-native populations here studied indicated these are closely related to southeastern Mexican populations of *D. stramonium*, suggesting that Spanish non-native populations likely descend from Mexican native populations (Velázquez-Márquez, Personal comm., 2024). It is possible that some differences between native and non-native populations were already present among native source populations. Nonetheless, this scenario is unlikely since changes in defensive traits and mating system traits have been found in *D. stramonium* from the non-native habitats (Valverde et al. 2015; Jiménez-Lobato et al. 2018; Castillo et al. 2019). Since one of the main differences between the native and non-native habitats is the presence of herbivores (and pollinators), this suggests that ecological interactions (or its absence) are important in shaping the evolution of phenotypic differentiation between regions despite the original genetic makeup of non-native populations (Lin et al. 2015; Jiménez-Lobato et al. 2018).

We detected fitness cost of tolerance to leaf damage in both regions (Fig. 4). In non-native populations costs of tolerance were higher although not significantly different from native populations (Table S4). The presence of fitness cost for tolerance has important implication for its evolution in the non-native habitat. First, benefits for tolerance are “cashed” when damaged occurs. In Spain, *D. stramonium* is free from its specialist folivores and receive very low levels of damage (2% of total leaf area) by a generalist herbivores, *Helicoverpa armigera* (Valverde et al. 2015), suggesting that levels of resistance in non-native populations are sufficient to deter generalist herbivores which makes tolerance less beneficial. Nonetheless, simulations found that “cheap” tolerance inhibits resistance due trade-offs between these two defenses (Sandoval-Castellanos and Núñez-Farfán 2023). Thus, the presence of fitness cost for tolerance can explain why non-native populations did not evolve higher levels of tolerance. Second, fitness costs can be compensated whenever tolerance traits contribute to promote growth and/or competitive ability, and to other functions like the response to abiotic stresses (Herms and Mattson 1992; Müller-Schärer et al. 2004; Genton et al. 2005). This can complicate the formulation of accurate predictions about the direction in which tolerance in non-native populations might evolve. Finally, depending on the selective context, several scenarios are possible for the evolution of defenses of introduced plant populations (Núñez-Farfán and Valverde 2020), where EICA is just one of these scenarios (Honor and Colautti 2020) and the reduction of defenses is not a rule.

Tolerance to leaf damage can be attained by plastic responses after damage, although constitutive values of traits can contribute to the capacity of tolerating damage (Núñez-Farfán et al. 2007; Fornoni 2011). We detected an association for two plasticity foliar traits in this study, plasticity in leaf area, and plasticity in biomass allocated to leaves (LMF) (Table 1). The association was present in both experiments and the trait that explain most variance in tolerance was plasticity in LMF. There were differences between regions only for plasticity in leaf area, being higher in native populations (Fig S1, Table S1), although this difference was marginally significant in the second experiment (Table S2), and plasticity in leaf area explains a small amount of variation in tolerance (Table 1). In contrast, there were no differences in plasticity in LMF between native and non-native populations and this can explain why we found similar levels of plasticity in both regions; the underlying trait of tolerance (or fitness reaction norm) (sensu Alpert and Simms 2002) was not different between native and non-native populations. When examining the association between traits and tolerance separately for each region, we found other traits influencing tolerance, like biomass allocated to stems (SMF, higher in native populations) or leaf area, but like the analysis with both regions (Table 1), it is possible to observe that plasticity in foliar traits is related to tolerance. This would support the idea that the tolerance in *D. stramonium* is achieved at the leaf level (Fornoni and Núñez-Farfán 2000; Camargo et al. 2015; Cisneros-Silva et al. 2017), and this is maintained in the non-native area. It is likely that new conditions in the non-native area promoted changes in traits like SLA, RMF, and seed production, and not in the plasticity of traits that underlay tolerance. Since several traits can contribute to tolerance in non-native populations and can be different for each species (Meyer and Hull-Sanders 2008; Bossdorf et al. 2008), the study of the traits that underlies the tolerant response of plants can contribute to a better understanding of the evolution of tolerance in an environment free from specialist herbivores.

## Conclusion

Our results demonstrated that introduced and native population of *Datura stramonium* did not differ in tolerance to leaf damage. Traits like leaf area, SLA, and allocation to stems and roots had diverged between these populations, although the response to leaf damage (plasticity) that underlies tolerance remains the same. Also, contrary to the EICA hypothesis, the fitness value in absence of damage of non-native populations was lower than native populations. The evolution of defense in an environment free from adapted herbivores is more complex that the ERH and EICA hypothesis initially suggested, since there is differentiation between populations in traits that can contribute to competitive ability or growth but are not translated in higher fitness for the non-native populations.

### Supplementary Information

Below is the link to the electronic supplementary material.

Supplementary file1 (DOCX 535 KB)

## Data Availability

The data supporting this article have been uploaded to Figshare (10.6084/m9.figshare.26098636.v1).
