# Supplementary material for: Plasticity in biomass allocation underlies tolerance to leaf damage in native and non-native populations of Datura stramonium
Source: Oecologia. 2024 Jul 24;205(3-4):613–26. doi: 10.1007/s00442-024-05585-0 (PMC11358249; doi:10.1007/s00442-024-05585-0)
Supplement: Supplementary file 1 — Supplementary file1 (DOCX 535 KB) [file 442_2024_5585_MOESM1_ESM.docx]

**Electronic supplemental material 1 (ESM1)**

Plasticity in biomass allocation underlies tolerance to leaf damage in native and non-native populations of *Datura stramonium*



Franco Liñán-Vigo^1^, Juan Núñez-Farfán^1^

^1^ Laboratorio de Genética Ecológica y Evolución, Departamento de Ecología Evolutiva, Instituto de Ecología. Universidad Autónoma de México, Circuito Exterior, Ciudad Universitaria, 04510 México, Ciudad de México, México

*Corresponding author: Juan Núñez-Farfán, email: farfan@unam.mx

**Table S1** Nested ANOVAs for *plasticity* of traits from the first experiment. Significant effects (*P*<0.05) in bold letters.

| **Trait** | **Factor** | **DF** | **SS** | **MS** | ***F*** | ***P-*value** |
| --- | --- | --- | --- | --- | --- | --- |
| Leaf area | **Region** | 1 | 0.51 | 0.51 | 15.35 | 0.0002 |
|  | **Population (Region)** | 2 | 0.54 | 0.27 | 8.22 | 0.0007 |
|  | Error | 62 | 2.05 | 0.03 |  |  |
| Biomass | Region | 1 | 0.05 | 0.05 | 0.81 | 0.3703 |
|  | Population (Region) | 2 | 0.04 | 0.02 | 0.32 | 0.7308 |
|  | Error | 62 | 3.64 | 0.06 |  |  |
| Reproductive | Region | 1 | 0.01 | 0.01 | 0.17 | 0.6774 |
| mass | **Population (Region)** | 2 | 0.42 | 0.21 | 3.38 | 0.0405 |
|  | Error | 62 | 3.82 | 0.06 |  |  |
| Seed number | Region | 1 | 0.00 | 0.00 | 0.01 | 0.9414 |
|  | **Population (Region)** | 2 | 0.85 | 0.43 | 3.72 | 0.0299 |
|  | Error | 62 | 7.10 | 0.11 |  |  |
| LMF | Region | 1 | 0.00 | 0.00 | 0.02 | 0.8921 |
|  | Population (Region) | 2 | 0.40 | 0.20 | 2.13 | 0.1276 |
|  | Error | 62 | 5.84 | 0.09 |  |  |
| SMF | Region | 1 | 0.00 | 0.00 | 0.15 | 0.7027 |
|  | Population (Region) | 2 | 0.01 | 0.01 | 0.37 | 0.6926 |
|  | Error | 62 | 1.15 | 0.02 |  |  |
| RMF | Region | 1 | 0.05 | 0.05 | 0.75 | 0.3895 |
|  | Population (Region) | 2 | 0.20 | 0.10 | 1.46 | 0.2411 |
|  | Error | 62 | 4.34 | 0.07 |  |  |
| Reproductive | Region | 1 | 0.07 | 0.07 | 1.61 | 0.2090 |
| effort | Population (Region) | 2 | 0.13 | 0.07 | 1.58 | 0.2140 |
|  | Error | 62 | 2.57 | 0.04 |  |  |

**Table S2** Nested ANOVAs for *plasticity* of traits from the second experiment. Significant effects (*P*<0.05) in bold letters. Marginal significance (0.05<*P<0.1*) indicated by italics.

| **Trait** | **Factor** | **DF** | **SS** | **MS** | ***F*** | ***P*-value** |
| --- | --- | --- | --- | --- | --- | --- |
| Leaf area | *Region* | 1 | 0.16 | 0.16 | 3.41 | 0.0734 |
|  | *Population (Region)* | 2 | 0.25 | 0.13 | 2.71 | 0.0809 |
|  | Error | 34 | 1.59 | 0.05 |  |  |
| Biomass | Region | 1 | 0.03 | 0.03 | 0.81 | 0.3752 |
|  | Population (Region) | 2 | 0.09 | 0.04 | 1.13 | 0.3363 |
|  | Error | 34 | 1.34 | 0.04 |  |  |
| SLA | *Region* | 1 | 0.31 | 0.31 | 3.65 | 0.0645 |
|  | Population (Region) | 2 | 0.00 | 0.00 | 0.02 | 0.9762 |
|  | Error | 34 | 2.93 | 0.09 |  |  |
| LMF | Region | 1 | 0.00 | 0.00 | 0.01 | 0.9087 |
|  | Population (Region) | 2 | 0.04 | 0.02 | 0.50 | 0.6132 |
|  | Error | 34 | 1.30 | 0.04 |  |  |
| SMF | Region | 1 | 0.01 | 0.01 | 0.42 | 0.5229 |
|  | Population (Region) | 2 | 0.01 | 0.01 | 0.38 | 0.6901 |
|  | Error | 34 | 0.60 | 0.02 |  |  |
| RMF | Region | 1 | 0.03 | 0.03 | 0.48 | 0.4930 |
|  | Population (Region) | 2 | 0.11 | 0.06 | 1.03 | 0.3686 |
|  | Error | 34 | 1.86 | 0.05 |  |  |
| Seed number | Region | 1 | 0.00 | 0.00 | 0.04 | 0.8502 |
|  | Population (Region) | 2 | 0.28 | 0.14 | 2.16 | 0.1305 |
|  | Error | 34 | 2.22 | 0.07 |  |  |
| Seed mass | Region | 1 | 0.01 | 0.01 | 0.15 | 0.7049 |
|  | **Population (Region)** | 2 | 0.34 | 0.17 | 3.66 | 0.0362 |
|  | Error | 34 | 1.56 | 0.05 |  |  |
| Seed mean | Region | 1 | 0.00 | 0.00 | 0.07 | 0.7973 |
| mass | Population (Region) | 2 | 0.06 | 0.03 | 1.48 | 0.2415 |
|  | Error | 34 | 0.69 | 0.02 |  |  |

**Table S3** Traits related to fitness (seed number) in absence of damage (control treatment) in each experiment. The relationships were obtained after a stepwise regression (entrance level of *P* = 0.15, stay level of *P* = 0.10) where the response variable was seed number in absence of damage and the response variable was the other vegetative traits. The analysis was run for each region and combining them

|  | Region | Trait | Estimate | Cum. R^2^ | *P*-value |
| --- | --- | --- | --- | --- | --- |
| Experiment 1 |  | Leaf area | 0.452 | 0.192 | 0.0001 |
| Experiment 2 |  | Stem mass fraction | 0.574 | 0.127 | 0.0006 |
|  |  | Leaf area | 0.452 | 0.281 | 0.0056 |
| Experiment 1 | Native | Not significant | - | - | - |
|  | Non-native | Leaf area | 0.451 | 0.251 | 0.0062 |
|  |  | Leaf mass fraction | -0.282 | 0.315 | 0.072 |
| Experiment 2 | Native | Stem mass fraction | 0.511 | 0.161 | 0.0365 |
|  |  | Leaf area | 0.528 | 0.288 | 0.0682 |
|  | Non-native | Leaf area | 0.558 | 0.178 | 0.0364 |

**Table S4** Cost of tolerance (correlation coefficient) in native and non-native populations of *Datura stramonium* in two experiments.

|  | Native populations | Non-native populations | *z* value |
| --- | --- | --- | --- |
| Experiment 1 | -0.498 (n=37) | -0.717(n=29) | -1.362 (*P* =0.173) |
| Experiment 2 | -0.414 (n=18) | -0.682 (n=20) | -1.108 (*P* =0.268) |


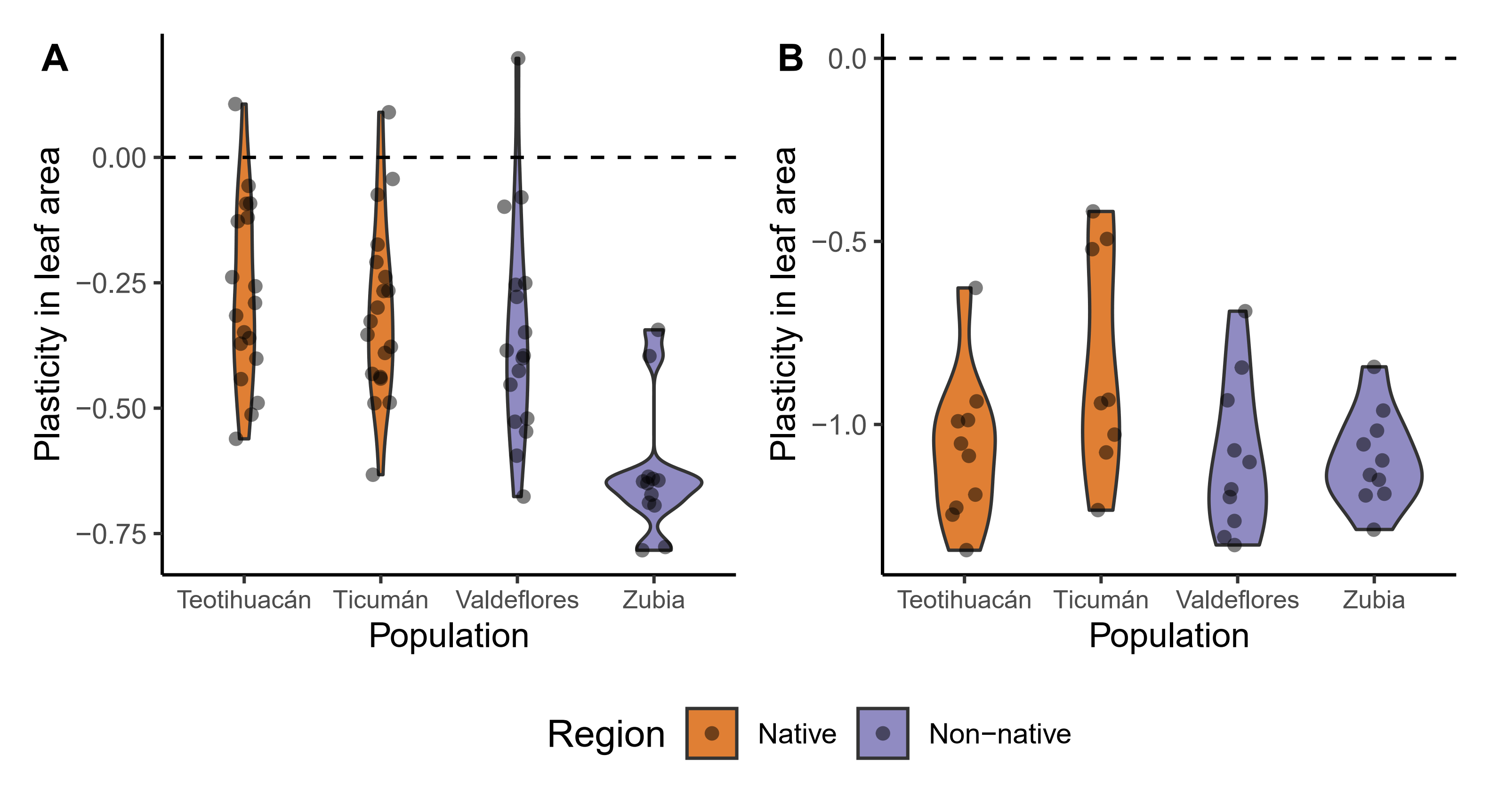


**Figure S1** Plasticity in leaf area for native Mexican populations and non-native Spanish populations of *Datura stramonium*. A. values for experiment 1. B. values for experiment 2.


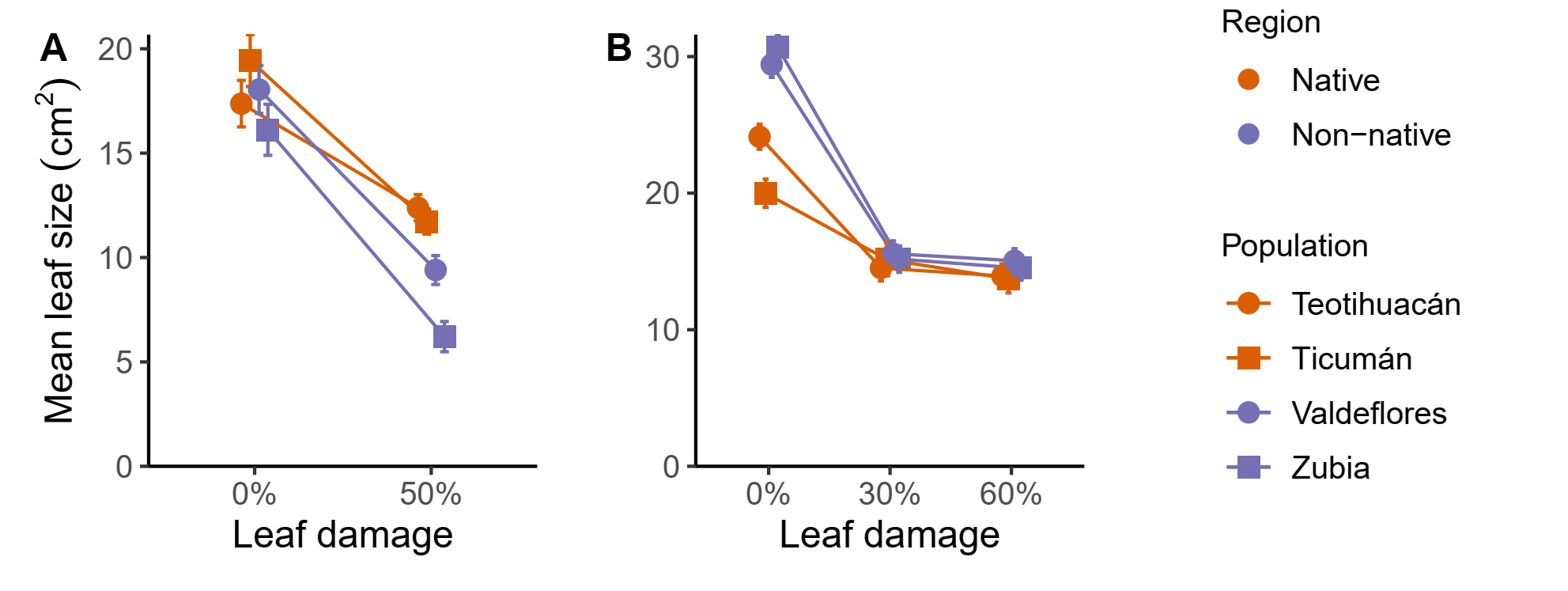


**Figure S2** Mean leaf size of plants of native and non-native populations of *D. stramonium.* Least square means and one standard error are represented. A. values for experiment 1. B. values for experiment 2. Native Mexican populations: Teotihuacán and Ticumán, Non-native Spanish populations: Valdeflores and Zubia.

**Table S5** Range for tolerance values found in previous studies.

| Study | Species | Tolerance measurement | Tolerance range |
| --- | --- | --- | --- |
| Croy et al. 2020 | *Phragmites australis* | log response ratio | approximately from -1.0 to 0.5 |
| Fornoni et al. 2003 | *Datura stramonium* | slope | from - 1.6 to 0.5 |
| Garrido et al. 2010 | *Datura stramonium* | slope | approximately from -0.7 to 0.2 |
| Giraldo 2015 | *Datura stramonium* | slope | approximately from -0.6 to 0.6 |
| Vargas-Ortiz 2015 | *Amaranthus cruentus* | slope | approximately from -0.8 to -0.1 |
| Wise et al. 2008 | *Solanum carolinense* | slope* | approximately from -0.4 to 0.7 |
| Experiment 1 | *Datura stramonium* | slope | from -0.6 to 0.8 |
| Experiment 2 | *Datura stramonium* | slope | from -0.5 to 0.6 |

*Estimated from reaction norms (Fig.4 in Wise et al. 2008)

***References***

Croy JR, Meyerson LA, Allen WJ, et al (2020) Lineage and latitudinal variation in *Phragmites australis* tolerance to herbivory: implications for invasion success. Oikos 129:1341–1357. <https://doi.org/10.1111/oik.07260>

Fornoni J, Valverde PL, Núñez-Farfán J (2003) Quantitative genetics of plant tolerance and resistance against natural enemies of two natural populations of. Evolutionary Ecology Research 5:1049–1065

Garrido E, Bennett AE, Fornoni J, Strauss SY (2010) Variation in arbuscular mycorrhizal fungi colonization modifies the expression of tolerance to above‐ground defoliation. Journal of Ecology 98:43–49. <https://doi.org/10.1111/j.1365-2745.2009.01586.x>

Giraldo Kalil LJ (2015) Efecto del sistema de apareamiento de *Datura stramonium* en las estrategias de defensa contra herbívoros. Tesis de Maestría, UNAM

Vargas-Ortiz E, Délano-Frier JP, Tiessen A (2015) The tolerance of grain amaranth (*Amaranthus cruentus* L.) to defoliation during vegetative growth is compromised during flowering. Plant Physiology and Biochemistry 91:36–40. <https://doi.org/10.1016/j.plaphy.2015.03.007>

Wise MJ, Cummins JJ, De Young C (2008) Compensation for floral herbivory in *Solanum carolinense*: identifying mechanisms of tolerance. Evol Ecol 22:19–37. <https://doi.org/10.1007/s10682-007-9156-x>
